# Effects of Brown Algae (*Laminaria japonica*) Extract on Growth Performance, Immune Function and Intestinal Health of Largemouth Bass (*Micropterus salmoides*)

**DOI:** 10.3390/ani15050622

**Published:** 2025-02-20

**Authors:** Jiajia Shen, Hongxiang Liu, Mengran Wang, Bo Lu, Ke Ke, Yunyong Wei, Feng Gao, Qiaozhen Wang, Shushi Huang, Yanqun Ma

**Affiliations:** 1 Key Laboratory of Aquatic Healthy Breeding and Nutrition Regulation of Guangxi Universities, College of Animal Science and Technology, Guangxi University, Nanning 530004, China; 2 Guangxi Key Laboratory of Marine Natural Products and Combinatorial Biosynethesis Chemistry, Guangxi Academy of Sciences, Nanning 530007, China; 3 National Key Laboratory of Non-Food Biomass Energy Technology, National Engineering Research Center for Non-Food Biorefinery, Institute of Biological Science and Technology, Guangxi Academy of Sciences, Nanning 530007, China

**Keywords:** *Micropterus salmoides*, brown algae extract, growth, intestinal flora

## Abstract

This study used largemouth bass (initial average weight: 33.33 ± 1.8 g) to explore the effects of adding different brown algae extracts to feed on the fish’s growth, immunity and intestinal health. Six groups were set up: a control (Group A), 0.1% sodium alginate (Group B), 0.1% oligotriosaccharide I (Group C), 0.1% oligotriosaccharide II (Group D), 0.2% brown algae powder (Group E) and 0.2% brown algae powder enzymatic product (Group F), with three replicates of 35 fish each, and a 56-day feeding experiment. Results: Compared to Group A, Groups C, D and F had a higher specific growth rate and lower feed coefficient (*p* < 0.05). Group D had enhanced serum SOD activity; Group F had increased antioxidant enzyme activity and decreased MDA content (*p* < 0.05). All experimental groups had higher serum LZM levels (*p* < 0.05), with no IgM difference (*p* > 0.05). In the intestine, treatment groups had higher α-amylase activity (*p* < 0.05) and no lipase difference (*p* > 0.05), and Groups C, D and F had higher trypsin activity (*p* < 0.05). Group F had the tallest villi, Group B had the thickest muscular layer (*p* < 0.05), and villus width was similar among groups (*p* > 0.05). The experimental groups had fewer intestinal pathogenic bacteria, and Group F had improved intestinal microorganism diversity and richness (*p* < 0.05). In conclusion, adding 0.1% oligotriosaccharide and 0.2% brown algae powder enzymatic product to feed can promote largemouth bass growth, antioxidant capacity and immunity. The 0.2% brown algae powder enzymatic product is better for intestinal development and flora improvement.

## Simple Summary

Given the expansion of largemouth bass farming and growing concerns about fish gut health, exploring beneficial feed additives is crucial. In this study, we used five brown algae extracts (sodium alginate, oligo trisaccharide I, oligo trisaccharide II, brown algae powder, and its enzymatic product) as feed additives for largemouth bass. By analyzing their growth performances, immune functions, and intestinal health, we identified the optimal additive. The results showed that adding 0.1% oligotrisaccharide I, 0.1% oligotrisaccharide II, and 0.2% brown algae powder enzymatic product promoted growth and enhanced antioxidant and immune abilities. The 0.2% brown algae powder enzymatic product was especially good for intestinal development and flora improvement, creating the potential for healthy aquaculture and better feed development.

## 1. Introduction

Largemouth bass (*Micropterus salmoides*), also known as California bass, is native to North America and belongs to Perciformes [1]. It is economically important in North America, Europe and China. In China, its farming areas have expanded from the coast to inland [2]. However, large-scale, high-density farming and overeating stress the fish, harming their gut health [3]. Therefore, in the current aquaculture system, finding feed additives for healthier largemouth bass guts is urgent. 

Brown algae constitutes a major type of economically cultivated algae in China. It serves as an excellent source for obtaining natural products and has a substantial output. It has nutrients like proteins, vitamins and minerals and active substances such as fucoidan, polysaccharides and polyphenols [4]. As the aquaculture industry develops and feed antibiotic use is restricted, brown algae extracts have drawn attention. Alginate, a key part of large marine brown algae cell walls, has antibacterial, anti-inflammatory and antioxidant functions [5]. However, its high water-soluble viscosity and large molecular weight limit its effects. The functional oligosaccharides derived from the degradation of alginate overcome the shortcomings of previous antibiotics, probiotics and enzyme preparations [6]. Moreover, they can enhance the disease resistance of animal bodies and promote their growth [7] and are beneficial for alleviating intestinal inflammation in mammals [8]. Some studies have discovered that adding brown algae extracts to feed can enhance the immunity of aquatic animals, improve intestinal morphology, significantly increase the height of long villi, induce the proliferation of intestinal flora and exert a positive effect on maintaining the bacterial diversity in the intestinal tract of fish and safeguarding intestinal health [7,9,10]. Adding brown algae polyphenol extracts to ruminant feed has many benefits. It can regulate the structure of the rumen flora, optimize the rumen digestion environment, effectively inhibit protein decomposition and significantly improve the in vitro digestibility of the feed [11]. 

As aquaculture aims for sustainability and high-quality growth, these extracts with rich bioactive substances have drawn much attention. However, most current studies focus on preparation methods, with scarce research into applications, especially in aquatic feeds. For the commercially important largemouth bass, application research into brown algae extracts is even rarer. To fill this gap, our experiment added five brown algae extracts (sodium alginate, oligo trisaccharide I, oligo trisaccharide II, brown algae powder and its enzymatic product) to largemouth bass feed. Over 56 days, we measured growth, serum, intestinal morphology and microbiota. The goal is to clarify how these extracts affect largemouth bass growth, immunity and intestinal health. The results could offer a theoretical basis for healthy cultivation, help develop better feed additives and boost the aquaculture industry. This research thus bridges a knowledge gap and shows the potential of brown algae extracts in largemouth bass aquaculture. 

## 2. Materials and Methods

### 2.1. Experimental Feed

(1) The sodium alginate used was a commercially available food-grade product, with an alginic acid content ≥ 95%. The raw material for production was kelp (*Laminaria japonica*). 

(2) Oligotrisaccharide I was prepared using highly active alginate lyase I developed by the research group (Guangxi Academy of Sciences, Nanning, China) to specifically hydrolyze food-grade sodium alginate. (The highly active alginate lyase I is patented in China. The optimal pH for the enzymatic hydrolysis reaction is 7.5, and the optimal temperature is 25 °C). The main component is brown algae oligosaccharide trisaccharide, accounting for 70%; the remaining components are disaccharide, tetrasaccharide and pentasaccharide. 

(3) Oligotrisaccharide II was prepared by hydrolyzing food-grade sodium alginate with alginate lyase II (Weilan Biotechnology Co., Ltd., Binzhou, China). Its main components are 25% disaccharide, 40% trisaccharide and 23% tetrasaccharide. 

(4) Brown algae powder was prepared by crushing commercially available dried kelp (*Laminaria japonica*) slices and passing them through a 200-mesh sieve. Its main components are alginate (content about 50%), mannitol, laminarin, fucose, crude protein, cellulose, vitamins, amino acids and various macro and trace elements. 

(5) Brown algae powder enzymatic product was prepared by qualitatively hydrolyzing brown algae (brown algae powder) with highly active alginate lyase I (Guangxi Academy of Sciences, Nanning, China), converting the alginic acid polysaccharides in the brown algae into oligosaccharides and simultaneously facilitating the dissolution of the various effective active components in the brown algae. The components of the algal oligosaccharides in the enzymatic hydrolysis products were detected as monosaccharides (2.31%), disaccharides (13.9%), trisaccharides (76.62%) and tetrasaccharides (7.17%) through thin-layer chromatography and ESI-MS analysis. 

A total of 6 experimental groups were set up in the experiment. The control group was A, and the other 5 groups were coded as B–F in sequence. Each group had 3 replicates. The feed formula was designed (Table 1). Based on the alginate content, 0.1% sodium alginate, 0.1% oligo trisaccharide I, 0.1% oligo trisaccharide II, 0.2% brown algae powder and 0.2% brown algae powder enzymatic product were successively added to the largemouth bass feeds of Groups B–F. All the added brown algae extracts, which were in powder form and soluble in water but insoluble in organic solvents, replaced the wheat flour in the basic formula. All raw materials were crushed and passed through a 40-mesh sieve. After accurate weighing, they were mixed uniformly. The mixing was carried out step by step from small to large according to the component proportions. Pellet feed with a diameter and length of 2.5 mm was made. Then the feed pellets were put into a constant-temperature drying oven and dried at 50 °C. After cooling, they were put into sealed bags and stored at −20 °C for later use. 

### 2.2. Aquaculture Management

Largemouth bass (initial average weight: 33.33 ± 1.8 g) were obtained from Guangxi Fangcao Angui Biotechnology Co., Ltd. (Nanning, China). The fish were cultured in rectangular tanks during the experimental period. Largemouth bass were fed with a commercial diet (Rongchuan, Zhuhai Co., Ltd., Guangzhou, China) for two weeks (the same as the experimental conditions) before being fed the experimental diets. A total of 630 fish were randomly assigned to 6 groups (35 fish per tank with 3 replicates in each group). They were fed regularly twice daily (7:30 a.m., 18:30 p.m.) until apparent satiation. All uneaten feed pellets were collected, dried and weighed at 65 °C. Aerated tap water served as the source of water for aquaculture. The feeding trial lasted 56 days. All experimental groups were measured using a water quality analysis kit (Nanhua Qianmu Biotechnology Co., Ltd., Dongguan, China), with a detection frequency of three times a week. The experimental conditions were as follows: water temperature, 29–30 °C; pH = 7.6–7.8; dissolved oxygen, 6–9 mg/L; ammonia nitrogen < 0.1 mg/L; nitrite nitrogen < 0.1 mg/L. 

### 2.3. Sampling

Before sampling, all largemouth bass were fasted for 24 h and then anesthetized with tricaine methanesulfonate (MS-222) at a dose of 55 mg/L. The body weight and length of the fish were measured. Subsequently, all fish were sampled and sacrificed to measure their individual physiology. The blood samples were quickly collected from the tail vein into Eppendorf tubes, incubated overnight at 4 °C and centrifuged at 4 °C and 3000× *g* for 10 min. The supernatant was transferred into new Eppendorf tubes and stored at −80 °C for subsequent analysis. Specimens were dissected on a sterile bench and weighed. The visceral weight and hepatopancreas weight were recorded. Twenty fish per replicate were dissected under sterile conditions to pull out the intestine. After that, about 4 cm of the anterior intestine was cut and fixed with paraformaldehyde solution to prepare intestinal tissue sections. Finally, the intestinal contents were collected and placed in a 1.5 mL sterile tube without RNA enzymes (four samples (each sample contains 5 fish) in each tank). After freezing in liquid nitrogen, they were stored in a −80 °C refrigerator for microbiome sequencing. 

### 2.4. Determination and Calculation

#### 2.4.1. Growth Indicators

Weight gain rate (WGR) = (Wt − W0)/W0 × 100%

Specific growth rate (SGR) = (lnWt − lnW0)/t × 100%

Feed conversion rate (FCR) = WF/WG

Hepatopancreas somatic indices (HSI) = Wh/W × 100%

Viscerosomatic index (VSI) = Wv/W × 100%

Condition factor (CF) = W/L^3^ × 100

Survival rate (SR) = Nt/N0 × 100%

In the formula, W0 is the initial body weight (g), Wt is the final body weight (g), t is the experimental duration (d), WF is the dry weight of the feed intake (g), WG is the weight gain (g), Wh is the liver weight (g), W is the body weight (g), Wv is the visceral mass weight (g), L is the body length (cm), Nt is the number of final fish (tail), and N0 is the number of initial fish (tail). 

#### 2.4.2. Non-Specific Immune Indicators

The levels of lysozyme (LZM), alkaline phosphatase (AKP), reduced glutathione (GSH), superoxide dismutase (SOD), malondialdehyde (MDA) and immunoglobulin M (IgM) in serum were determined using the enzyme-linked immunosorbent assay kit from Shanghai Yuanxin Biotechnology (Shanghai, China) according to the instructions. The mass concentrations of complement component 3 (C3) and catalase (CAT) were determined using enzyme-linked immunosorbent assay kits from Nanjing Jiancheng Bioengineering Institute (Nanjing, China). The operations were carried out according to the steps described in the kit operation manual. First, we prepared by equilibrating the kit, making buffers and processing samples. Then, we added standards and samples to the microplate. These were incubated to allow for antigen–antibody binding, and we added stop solution. We washed the plate multiple times to remove unbound substances. Next, we added the enzyme conjugate and incubated again, followed by another wash. After that, we added the color substrate for color development and finally added the stop solution and measured the absorbance with a microplate reader to calculate the sample concentrations from the standard curve. 

#### 2.4.3. Intestinal Digestive Enzymes

Lipase (LPS), trypsin (TPS) and α-amylase (AMS) were determined using micro-method kits from Shanghai Yuanxin Biotechnology (Shanghai, China). We homogenized the fish intestinal tissue, centrifuged it and took the supernatant as the sample. Then, we equilibrated the kit at room temperature and prepared the working solutions. Next, we added the standards and samples to a 96-well microplate, added the substrate working solution and incubated the microplate. Finally, we added the stop solution, measured the absorbance value with a microplate reader and calculated the activity or concentration of protease in the sample as well as the actual activity or content of protease in the fish intestinal tissue through the standard curve (we followed the specific steps as described in the instruction manual). 

#### 2.4.4. Intestinal Tissue Sections

The midgut samples were entrusted to Wuhan Servicebio Technology Co., Ltd. (Wuhan, China) for paraffin embedding and Hematoxylin and Eosin (HE) staining to make tissue sections. After scanning and imaging with the PANNORAMIC DESK panoramic section scanner (3DHISTECH, Budapest, Hungary), the villus height, villus width, crypt depth and intestinal wall thickness were determined using Image-Pro Plus 6.0 analysis software. 

#### 2.4.5. Intestinal Microbial Sequencing

The genomic DNA of the midgut microbiota was entrusted for extraction by Guangzhou Genedenovo Biotechnology Co., Ltd. (Guangzhou, China) The primer sequences 341F: CCTACGGGNGGCWGCAG and 806R: GGACTACHVGGGTATCTAAT were designed for the V3–V4 region fragment of the bacterial 16S rDNA gene. The purified amplified products, namely amplicons, were ligated with sequencing adapters to construct a sequencing library and subsequently subjected to sequencing and analysis on the Illumina platform (Illumina, Inc., San Diego, CA, USA). Bioinformatic analysis was performed using Omicsmart, a dynamic real-time interactive online platform for data analysis (http://www.omicsmart.com, accessed on 2 November 2024). We calculated the OUT quantity and Alpha diversity indices such as Sobs, Shannon, Simpson, Chao1, ACE, Good’s coverage, etc. Meanwhile, we statistically analyzed the species abundance of each sample at the two taxonomic levels of phylum and genus. 

### 2.5. Data Analysis

The results were analyzed using SPSS 26 statistical software. Statistical analysis was performed by one-way ANOVA and Duncan’s multiple range test. Differences with *p* < 0.05 were regarded as statistically significant. The data results were all expressed as “mean ± standard deviation”. 

## 3. Results

### 3.1. The Effects of Adding Brown Algae Extract to Feed on the Growth of Largemouth Bass

It can be concluded from Table 2 that at the end of the feeding experiment, the SGR of Groups D and F was significantly higher than that of the control group (*p* < 0.05), increasing by 30% and 44%, respectively; the SGR of Groups C, D, E and F was significantly lower than that of the control group (*p* < 0.05), decreasing by 1.44, 1.28, 0.71 and 1.66, respectively. The SR of group B was the lowest, significantly lower than that of other groups. The SR of groups D and F was the highest, being 93.33% and 94.44%, respectively; the CF of group F was the highest, 0.28 g/cm^3^ higher than that of the control group (*p* < 0.05); there was no significant difference in VAI among groups (*p* > 0.05). 

### 3.2. The Effect of Brown Algae Extract Addition to Feed on Largemouth Bass Serum Antioxidation

As in Table 3, serum GSH levels were similar between the experimental and control groups (*p* > 0.05). SOD levels in Groups D and F were notably higher than the control (*p* < 0.05), hitting 55.43 U/mL and 54.83 U/mL. MDA levels in Group C were much higher than in the control (*p* < 0.05). Group F had the lowest MDA content and the highest CAT content (*p* < 0.05). 

### 3.3. The Effect of Adding Brown Algae Extract to Feed on Largemouth Bass Serum Non-Specific Immunity

As in Table 4, compared to the control group, the experimental groups’ serum LZM content rose significantly (*p* < 0.05), with Group D having the highest at 29.58 ug/mL. Group F had the highest AKP content, significantly higher than others (*p* < 0.05). Compared to Group A, AKP in Group B and E increased by 3.09 and 3.16 king unit/100 mL, respectively. Each experimental group’s serum complement C3 content increased. C3 in Groups B, D and F rose significantly (*p* < 0.05), by 7.64, 7.98, 6.41 ug/mL, respectively. Serum IgM content showed no significant difference among all groups (*p* > 0.05). 

### 3.4. The Effect of Adding Brown Algae Extract to Feed on Largemouth Bass Intestinal Digestive Enzymes

It can be discerned from Table 5 that, in contrast to the control group, the AMS content of the experimental groups rose conspicuously (*p* < 0.05). Among them, the AMS content of Group B was the highest, attaining 0.41 ug/mL. Nevertheless, there was no marked disparity in LPS content among all groups (*p* > 0.05). The TPS content of Groups C, E and F was significantly higher than that of the control group *(p* < 0.05), with increments of 2.37 U/g, 3.28 U/g and 1.95 U/g, respectively. 

### 3.5. The Effect of Adding Brown Algae Extract to Feed on Largemouth Bass Intestinal Histomorphology

**Figure 1:**
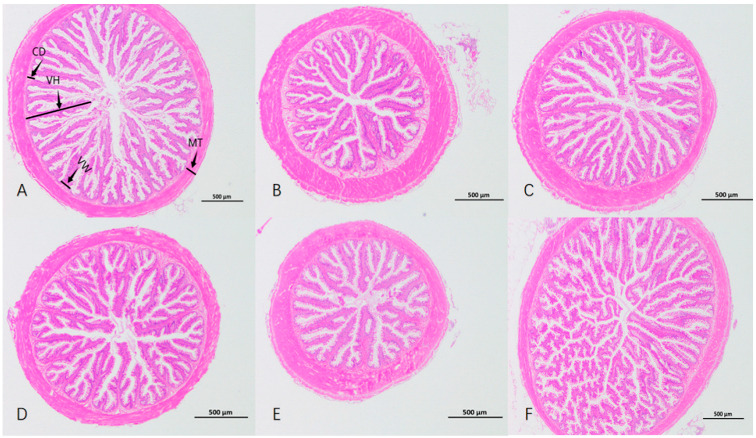
Intestinal sections of *Micropterus salmoides* with different brown algae extracts added in the feed. VH: villus height; VW: villus width; CD: crypt depth; MT: muscular layer thickness. (**A**–**F**) represent groups.

As depicted in Table 6 and Figure 1, the VH of Group F was the highest (*p* < 0.05), conspicuously higher than that of the other groups. There was no significant variance in VW among all groups (*p* > 0.05). The intestinal CD and MT of Group B were significantly higher than those of the control group (*p* < 0.05). The VH/CD of Group A was significantly higher than that of the control group (*p* < 0.05). 

### 3.6. The Effect of Adding Brown Algae Extract to Feed on the Intestinal Microbiota of Largemouth Bass

#### 3.6.1. Intestinal α-Diversity

A total of 992,485 pairs of raw reads and 991,582 pairs of clean reads were obtained from the sequencing of 18 samples, and an average of 55,087 clean reads were generated for each sample. Group F’s Sobs index was significantly higher than the control group’s (*p* < 0.05). There were no significant differences in the Chao1, ACE and Good’s coverage indices among all groups (*p* > 0.05). The experimental groups showed no significant differences in the Shannon and Simpson indices compared with the control group (*p* > 0.05) (Table 7).

#### 3.6.2. Analysis of Species Composition of Intestinal Microorganisms

**Figure 2:**
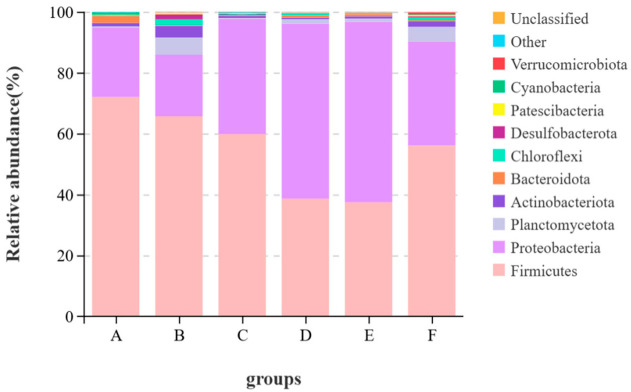
Stacked graph of species at the phylum level.

It can be seen from Figure 2 that at the phylum level, the top ten phyla by average abundance in all samples were *Firmicutes*, *Proteobacteria*, *Planctomycetota*, *Actinobacteriota*, *Bacteroidota*, *Chiloroflexi*, *Desulfobacterota*, *Patescibacteria*, *Cyanobacteria* and *Verrucomicrobiota*. Other known species are classified as “others”, and unknown species are marked as “unclassified”. The dominant phyla of the largemouth bass were *Firmicutes* and *Proteobacteria*. The relative abundance of *Firmicutes* in the control group was the highest, reaching 71.9%. Compared with the control group, the relative abundances of *Firmicutes* in Groups B, C, D, E and F all decreased by 6.28%, 12.15%, 33.33%, 34.49% and 15.92%, respectively. The relative abundance of *Cyanobacteria* in the control group was 0.68%, and the relative abundances of *Cyanobacteria* in the experimental groups were all less than 0.2%. The content of *Verrucomicrobiota* in Group F was the highest, which was 0.66% higher than that in the control group. 

**Figure 3:**
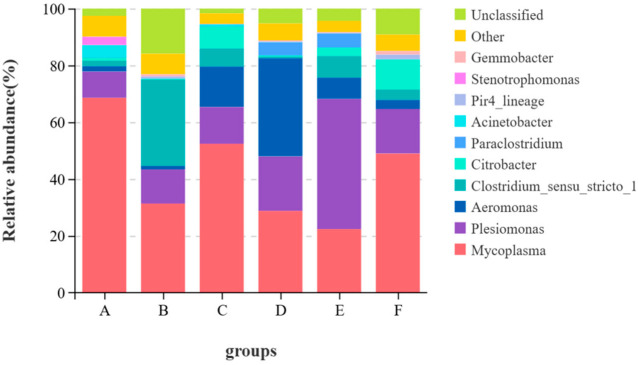
Stacked graph of species at the genus level.

From Figure 3, at the genus level, Mycoplasma and Plesiomonas were common dominant genera in all groups. Mycoplasma levels dropped in Groups B, C, D, E and F. Acinetobacter levels decreased in all test groups. Aeromonas levels rose in Groups C and D. 

## 4. Discussion

Brown algae extract benefits aquatic animal growth and shows great application and development potential in aquatic animal feed [12,13,14,15]. Research on *Takifugu obscurus* showed that brown algae oligosaccharides can promote its growth [14]. Adding 0.7 g/kg and 6.0 g/kg brown algae oligosaccharides to *Trachinotus ovatus’* diet significantly increased its WGR and SGR and reduced FCR [15]. Some studies have found that adding brown algae extract to feed enhanced *Oreochromis niloticus’* growth performance [16]. In this study, adding oligosaccharide trisaccharide I and II and enzymatic hydrolysate of brown algae powder to largemouth bass feed increased WGR and SGR and reduced FCR, similar to previous research. The FCR of the brown algae powder’s enzymatic hydrolysate group was as low as 0.84, suggesting that adding 0.2% of it to the feed can significantly improve the fish’s feed absorption and utilization. 

CF is an important indicator reflecting the nutritional status of fish [17]. Generally, the higher the CF is, the more material and energy are stored in the fish body [18]. Some studies have found that adding 6.0 g/kg of brown algae oligosaccharides to feed can significantly increase the CF of *Trachinotus ovatus* [15]. Adding mannan oligosaccharides to their feed significantly increased the CF of *Epinephelus lanceolatus* ♀ *× Epinephelus fuscoguttatus* ♂ [19]. In a study of juvenile *Micropterus salmoides*, adding laminarin polysaccharides to feed increased the CF of *Micropterus salmoides* [12]. In this study, the CF of the brown algae enzymatic hydrolysate group was significantly higher than that of the control group. This may be due to the brown algae enzymatic hydrolysate group having more brown algae oligosaccharides, along with mannan oligosaccharides and laminarin polysaccharides. 

Measuring serum antioxidant and immune enzyme activities can assess fish health [19]. Research shows brown algae extract’s functional oligosaccharides can regulate animal serum biochemical indices, boost antioxidant capacity and enhance non-specific immunity [20,21,22,23]. MDA, a lipid peroxidation end-product, reflects the body’s antioxidant potential and liver peroxidation damage [24]. In this experiment, the oligotriosaccharide I group had higher serum MDA and lower GSH levels. Since the liver synthesizes GSH, this indicates oxidative liver damage in largemouth bass. CAT and SOD are key fish antioxidant enzymes [25,26,27]. In the oligotriosaccharide II group, serum MDA decreased and GSH increased compared to the I group, possibly related to CAT and SOD levels. Studies show CAT can boost GSH activity by removing hydrogen peroxide from SOD-catalyzed superoxide anion disproportionation, inhibiting MDA formation [28,29,30,31]. Enhanced antioxidant enzyme activity slows lipid peroxidation and reduces tissue damage. Alginate oligosaccharides can enhance the antioxidant capacity of *Scophthalmus maximus* [32]. In this experiment, the serum antioxidant capacity of the oligotriosaccharide II group improved. However, the same did not happen in the oligotriosaccharide I group. Maybe the high concentration of oligotriosaccharides in the oligotriosaccharide I group damaged the liver. This needs further study. Compared with the control group, the brown algae powder enzymatic hydrolysate reduced largemouth bass’s serum MDA concentration by enhancing antioxidant enzyme activity and reducing lipid peroxide accumulation. 

LZM can hydrolyze the mucopolysaccharides in pathogenic bacteria into glycopeptides and promote the dissolution of bacteria. It has functions such as antibacterial, anti-inflammatory and antiviral [33]. In this trial, compared with the control group, the serum LZM content in the experimental group was significantly increased. At the same time, an increase in C3 content was also found. C3 is an important component of the fish complement system and plays an important role in the immune response of fish. It can not only play a bactericidal role in fish blood or mucus but also combine with specific sites on the surface of phagocytic cells to promote phagocytosis [34]. The increase of C3 in this trial might be due to the activation of phagocytic cells by the brown algae extract, promoting the synthesis of C3 [35]. However, not all experimental groups were significantly higher than the control group, possibly related to the instability of C3 [36]. The increase in the activities of LZM and C3 indicates the enhancement of the immune response of largemouth bass. AKP is an important type of enzyme for maintaining the health of fish and is directly involved in the transfer and metabolism of phosphate groups in the fish body [37]. In this trial, the content of AKP in the serum of the enzymatic hydrolysate of brown algae powder group was significantly higher than that of the control group. Similar reports on the effect of brown algae extract on AKP in aquatic animals have also been found in *Penaeus orientalis* [38], *Apostichopus japonicus* [39], *Scophthalmus maximu* [40], etc. AKP in Group F increased significantly. This may be due to functional oligosaccharides increasing the abundance of Verrucomicrobiota in the group’s intestines [41]. Verrucomicrobiota can produce short-chain fatty acids like propionic and butyric acid in the intestines. Butyric acid can significantly boost the expression of intestinal immune genes [42], promoting AKP activity increase. Immunoglobulin IgM showed no significant difference among groups. This means the brown algae extract added to the feed does not negatively affect largemouth bass’ IgM. In conclusion, the functional oligosaccharides in the brown algae extract help improve largemouth bass’ antioxidant capacity and enhance their immunity. Digestive enzyme activity is important in fish digestive physiology research [43]. α-amylase can hydrolyze starch into small-molecule oligosaccharides [44]. Ashouri, G et al. [45] found that adding brown algae oligosaccharides to barramundi (*Lates calcarifer*) feed increased intestinal lipase activity. A study [9] showed that adding different concentrations of brown algae oligosaccharides to turbot (*Scophthalmus maximus*) feed enhanced intestinal amylase and protease activities. In this study, adding brown algae extract to largemouth bass feed significantly increased intestinal α-amylase activity. 

The normal development of the intestinal structure in fish is important for digestion, absorption and immune function [46,47,48]. Intestinal villus height (VH), villus width (VW), crypt depth (CD) and muscular layer thickness (MT) can reflect the fish’s intestinal digestive and absorptive capacity. Generally, taller and wider villi mean a larger intestinal absorption area [49]. In this study, long villus width was similar among groups. The enzymatic hydrolysate of brown algae powder group had the highest villus height, extremely different from the control. Intestinal sections showed its villi were long and dense, with many branches and folds and a large absorption area. Villi in the brown algae powder group were short and sparse. Compared with the control group, the digestive performance decreased. The enzymatically hydrolyzed brown algae extract converted alginic acid polysaccharides into oligosaccharides, which was more conducive to nutrient digestion and absorption. One study [50] shows that adding oligosaccharides to feed benefits animal intestinal structure and increases villus height, similar to this study’s results. Intestinal peristalsis mainly relies on circular muscle contraction, so a thicker intestinal muscular layer can promote peristalsis and aid absorption. In this study, the sodium alginate group had short, sparse villi but a significantly thicker muscular layer.The likely reason is that sodium alginate has high viscosity [51] and is hard to digest, causing continuous peristalsis and passive structural changes for better food digestibility. This could be why largemouth bass in the sodium alginate group had a lower survival rate. Crypt depth shows the maturation rate of intestinal epithelial cells. These cells move and change from the crypt base to the villi, forming absorptive villus cells [52,53]. VH/CD reflects intestinal digestion and absorption. A higher ratio means a higher cell maturation rate and stronger nutrient absorption [53]. In this experiment, the VH/CD of the enzymatic hydrolysate of brown algae powder group was extremely significantly different from that of the control group. Combined with the fact that the WGR and SGR of this group were both significantly increased, this indicates that the enzymatic hydrolysis of brown algae powder enhanced the nutrient absorption and utilization of largemouth bass. To sum up, the enzymatic hydrolysate of brown algae powder contributes to the synthesis of intestinal digestive enzymes, improves the intestinal morphological structure of largemouth bass, promotes the growth of intestinal villi and facilitates the absorption of nutrients. 

The intestinal microorganisms of fish also play important roles in many aspects such as nutrient absorption, growth and development and immune defense [54,55]. There are many factors influencing the quantity and structure of intestinal flora in fish and the ingested feed is the most important one among them [56]. Therefore, the effect of brown algae extract on intestinal health can be measured by comparing the changes in intestinal flora. Studies have shown [12,45,57] that brown algae extract can change the intestinal flora of fish and regulate the immunity and growth of fish. In this study, the α-diversity index indicated that adding the enzymatic hydrolysate of brown algae powder to the feed significantly increased the diversity and richness of intestinal microorganisms, while the other groups showed no significant performance. This indicates that adding brown algae extract to feed does not disrupt the diversity of intestinal flora, and the enzymatic hydrolysate of brown algae powder has potential in regulating intestinal health. *Proteobacteria*, *Actinobacteria*, *Bacteroidetes*, *Fusobacteria*, *Firmicutes*, etc. are the main phyla in the gastrointestinal tract of fish [56]. *Firmicutes* can regulate the absorption of dietary fat in the intestinal tract, and *Proteobacteria* helps maintain homeostasis in the anaerobic environment of the intestinal tract. These two phyla play important roles in the nutritional metabolism of fish [58,59], and these two phyla were also the dominant phyla in this experiment. In this study, the relative abundance of *Firmicutes* in the control group was the highest, among which the relative abundance of *Mycoplasma* reached 68.58%. The immune system of fish infected with *Mycoplasma* would be damaged and other pathogenic lesions could develop rapidly, leading to aggravated diseases [60]. The relative abundance of *Mycoplasma* in the experimental groups was lower than that in the control group. From the perspective of food health and fish health, the feed with added brown algae extract has certain safety advantages. The genus *Aeromonas* under *Proteobacteria* is pathogenic. Studies have shown [61] that although performance varies greatly among different genera, it is often related to digestive tract infections in animals. In this trial, the relative abundance of *Aeromonas* in the oligotriosaccharide II group reached 35.54%, but the relative abundance of Aeromonas in the oligotriosaccharide I group was 14.22%. This might be related to other components of the oligotriosaccharide II group. *Acinetobacter* is an important opportunistic pathogen in the animal body. When the animal’s resistance declines, it is prone to infection. Among them, *Acinetobacter baumannii* can cause liver damage in eels [62]. Compared with the control group, the relative abundance of *Acinetobacter* in the experimental groups decreased to less than 1%, indicating that brown algae extract is beneficial to the intestinal health of largemouth bass. To sum up, brown algae extract can improve the intestinal flora of largemouth bass, reduce pathogenic bacteria and lower the risk of disease in largemouth bass. 

## 5. Conclusions

The extracts of these five types of brown algae (*Laminaria japonica*) have no negative impact on the growth and immune function of largemouth bass. Among them, 0.1% oligotriosaccharide I, 0.1% oligotriosaccharide II and 0.2% brown algae powder enzymatic product significantly promote the growth of juvenile largemouth bass. Based on the comprehensive analysis of various indicators, adding 0.2% brown algae powder enzymatic product to the feed can help promote the growth of larval largemouth bass, enhance their non-specific immune performance and improve the morphology of intestinal tissues and the structure of the intestinal flora, which is beneficial to intestinal health. 

## Figures and Tables

**Table 1:** Formulation and proximate composition of the experimental diets (g·(100 g)^−1^).

Ingredients	Group					
	A	B	C	D	E	F
Fishmeal	35.00	35.00	35.00	35.00	35.00	35.00
Wheat flour	20.00	19.90	19.90	19.90	19.80	19.80
Soybean meal	15.00	15.00	15.00	15.00	15.00	15.00
Peanut meal	15.00	15.00	15.00	15.00	15.00	15.00
Beer yeast	4.00	4.00	4.00	4.00	4.00	4.00
Fish oil	4.00	4.00	4.00	4.00	4.00	4.00
Soybean oil	4.00	4.00	4.00	4.00	4.00	4.00
Vitamin and mineral premix *	2.00	2.00	2.00	2.00	2.00	2.0
Ca(H_2_PO_4_)_2_	0.50	0.50	0.50	0.50	0.50	0.50
Choline chloride	0.50	0.50	0.50	0.50	0.50	0.50
Sodium alginate	0.00	0.10	0.00	0.00	0.00	0.00
Oligotrisaccharide I	0.00	0.00	0.10	0.00	0.00	0.00
Oligotrisaccharide II	0.00	0.00	0.00	0.10	0.00	0.00
Brown algae powder	0.00	0.00	0.00	0.00	0.20	0.00
Brown algae powder enzymatic product	0.00	0.00	0.00	0.00	0.00	0.20
Total	100	100	100	100	100	100
**Nutritional level**						
Crude protein	43.06	43.43	43.12	43.10	42.61	42.60
Crude lipid	8.70	8.75	8.50	9.10	9.45	9.90
Water content	11.90	12.90	13.60	12.10	12.60	12.20
Ash	96.30	96.80	97.40	96.90	96.70	95.00

* Purchased from Cangzhou Xindadi Biotechnology Co., Ltd. (Cangzhou, China). According to the instructions, 20 g of vitamin and mineral premix are to be added to every 1 kg of feed. The guaranteed values of product composition analysis are as follows: 10,000–500,000 IU of vitamin A and vitamin D3 per kilogram; 1–50 mg of vitamin B1, vitamin E and vitamin B2 per kilogram. The nutritional levels in the table are measured values.

**Table 2:** Effects of adding brown algae extract to feed on growth performance of *Micropterus salmoides*.

Index	Group					
	A	B	C	D	E	F
IBW (g)	33.37 ± 1.90	33.30 ± 0.00	33.93 ± 1.10	34.83 ± 1.30	33.74 ± 2.1	32.95 ± 1.95
FBW (g)	57.83 ± 1.52 ^c^	57.53 ± 1.07 ^c^	64.56 ± 3.75 ^bc^	71.48 ± 6.38 ^ab^	63.28 ± 4.58 ^c^	72.95 ± 3.91 ^a^
WGR (%)	52.2 ± 4.87 ^cd^	36.27 ± 6.41 ^d^	75.43 ± 8.73 ^bc^	92.6 ± 31.40 ^ab^	58.9 ± 16.91 ^cd^	109.19 ± 7.46 ^a^
SGR (%/d)	0.98 ± 0.10 ^c^	0.98 ± 0.033 ^c^	1.15 ± 0.13 ^abc^	1.28 ± 0.22 ^ab^	1.12 ± 0.19 ^bc^	1.42 ± 0.06 ^a^
FCR	2.52 ± 0.24 ^a^	2.79 ± 0.48 ^a^	1.08 ± 0.11 ^c^	1.24 ± 0.12 ^c^	1.81 ± 0.38 ^b^	0.86 ± 0.044 ^c^
SR (%)	87.7 ± 1.93 ^bc^	78.89 ± 3.85 ^d^	92.22 ± 1.93 ^ab^	93.33 ± 3.33 ^a^	84.44 ± 1.93 ^c^	94.44 ± 1.93 ^a^
VSI (%)	10.72 ± 1.01	10.20 ± 1.54	11.00 ± 1.07	9.42 ± 0.93	10.92 ± 0.10	11.37 ± 1.59
HSI (%)	4.36 ± 0.34 ^a^	3.52 ± 0.48 ^ab^	3.97 ± 0.14 ^ab^	3.03 ± 0.37 ^b^	3.76 ± 0.87 ^ab^	4.55 ± 0.87 ^a^
CF (g/cm^3^)	1.93 ± 0.13 ^b^	2.07 ± 0.06 ^ab^	2.04 ± 0.01 ^ab^	2.03 ± 0.17 ^ab^	2.02 ± 0.14 ^ab^	2.21 ± 0.10 ^a^

Different superscript letters within the same row indicate significant differences among groups (*p* < 0.05). IBW means the initial body weight; FBW means the final body weight; WGR means the weight gain rate; SGR means the specific growth rate; FCR means the feed conversation rate; SR means the survival rate; VSI means the viscerosomatic index. HSI means the hepatosomatic index; CF means the condition factor.

**Table 3:** Effects of adding brown algae extract to feed on serum antioxidation of *Micropterus salmoides*.

**Index**	**Group**					
	**A**	**B**	**C**	**D**	**E**	**F**
GSH (ug/mg)	3.64 ± 0.08 ^ab^	3.86 ± 0.06 ^ab^	3.20 ± 1.18 ^b^	4.57 ± 0.78 ^a^	4.57 ± 0.29 ^a^	4.19 ± 0.36 ^ab^
SOD (U/mL)	51.52 ± 0.53 ^b^	53.17 ± 0.67 ^ab^	54.46 ± 1.66 ^ab^	55.43 ± 2.69 ^a^	53.71 ± 0.31 ^ab^	54.83 ± 1.60 ^a^
MDA (nmol/mL)	2.54 ± 0.31 ^b^	2.86 ± 0.88 ^b^	3.91 ± 0.43 ^a^	2.69 ± 0.17 ^ab^	3.03 ± 0.74 ^b^	1.48 ± 0.41 ^c^
CAT (U/mL)	13.56 ± 0.50 ^c^	13.92 ± 1.43 ^c^	14.80 ± 0.08 ^c^	18.37 ± 0.29 ^b^	18.71 ± 0.23 ^b^	21.14 ± 0.13 ^a^

Different superscript letters within the same row indicate significant differences among groups (*p* < 0.05).

**Table 4:** Effects of adding brown algae extract to feed on *Micropterus salmoides* serum non-specific immunity.

Index	Group					
	A	B	C	D	E	F
LZM (μg/mL)	13.30 ± 0.59 ^c^	20.99 ± 1.25 ^b^	21.00 ± 1.34 ^b^	29.58 ± 0.76 ^a^	19.51 ± 0.64 ^b^	20.40 ± 1.20 ^b^
AKP (king unit/100 mL)	15.27 ± 1.48 ^cd^	18.36 ± 0.49 ^b^	16.38 ± 0.89 ^bc^	13.25 ± 1.47 ^d^	18.43 ± 1.15 ^b^	25.47 ± 0.823 ^a^
C3 (ug/mL)	33.89 ± 2.10 ^b^	41.53 ± 0.85 ^a^	35.27 ± 4.26 ^b^	41.87 ± 2.69 ^a^	35.00 ± 1.10 ^b^	40.30 ± 0.65 ^a^
IgM (ug/mL)	41.67 ± 4.76	41.13 ± 2.47	41.88 ± 4.52	43.80 ± 5.37	39.27 ± 0.92	40.33 ± 6.43

Different superscript letters within the same row indicate significant differences among groups (*p* < 0.05).

**Table 5:** Effects of adding brown algae extract to feed on *Micropterus salmoides* intestinal digestive enzymes.

Index	Group					
	A	B	C	D	E	F
LPS (U/g)	19.54 ± 1.08	20.61 ± 0.94	22.04 ± 1.08	21.08 ± 1.49	21.32 ± 2.17	22.28 ± 0.41
AMS (μg/mL)	0.06 ± 0.01 ^d^	0.41 ± 0.04 ^a^	0.15 ± 0.01 ^c^	0.29 ± 0.06 ^b^	0.17 ± 0.01 ^c^	0.30 ± 0.02 ^b^
TPS (U/g)	4.42 ± 0.87 ^c^	2.43 ± 0.30 ^d^	7.15 ± 0.65 ^ab^	1.24 ± 0.18 ^e^	7.70 ± 0.61 ^a^	6.37 ± 0.64 ^b^

Different superscript letters within the same row indicate significant differences among groups (*p* < 0.05).

**Table 6:** Morphological indicators of intestinal tissue of *Micropterus salmoides* (µm).

Index	Group					
	A	B	C	D	E	F
VH	707.94 ± 60.29 ^b^	505.28 ± 48.95 ^c^	650.18 ± 54.43 ^b^	624.57 ± 58.20 ^b^	500.75 ± 59.74 ^c^	1069.98 ± 130.22 ^a^
VW	91.03 ± 14.04	100.91 ± 28.08	91.59 ± 10.57	96.76 ± 18.53	74.86 ± 16.84	85.93 ± 19.98
CD	49.40 ± 19.50 ^b^	69.11 ± 18.19 ^a^	49.22 ± 11.37 ^b^	35.30 ± 5.83 ^bc^	43.28 ± 8.90 ^b^	23.01 ± 8.08 ^c^
MT	99.05 ± 14.51 ^d^	199.80 ± 20.81 ^a^	131.67 ± 40.23 ^c^	147.63 ± 23.18 ^bcd^	183.83 ± 28.68 ^ab^	136.36 ± 42.73 ^cd^
VH/CD	15.85 ± 4.63 ^bc^	7.63 ± 1.52 ^c^	13.80 ± 3.23 ^bc^	22.62 ± 8.96 ^b^	11.92 ± 2.49 ^c^	50.07 ± 13.04 ^a^

Different superscript letters within the same row indicate significant differences among groups (*p* < 0.05). VH means the villus height; VW means the villus width; CD means the crypt depth; MT means the muscular layer thickness; VH/CD means the ratio of villus height to crypt depth.

**Table 7:** The α-diversity of intestinal microorganisms of *Micropterus salmoides* in each group.

Index	Group					
	A	B	C	D	E	F
Sobs	196.5 ± 4.95 ^bc^	276 ± 22.63 ^ab^	174.5 ± 61.52 ^b^	204.5 ± 0.71 ^bc^	266.5 ± 10.61 ^abc^	256 ± 90.51 ^a^
Chao1	247.9 ± 41.45	307.61 ± 38.92	215.76 ± 31.88	237.91 ± 12.06	301.385 ± 2.64	276.085 ± 90.50
ACE	241.66 ± 32.97	303.365 ± 26.03	220.16 ± 33.28	236.335 ± 17.66	306.005 ± 1.46	287.625 ± 94.91
Shannon	2.29 ± 0.65 ^ab^	3 ± 0.61 ^ab^	2.435 ± 0.26 ^b^	2.715 ± 0.01 ^ab^	2.68 ± 0.04 ^ab^	2.755 ± 1.51 ^a^
Simpson	0.515 ± 0.11 ^ab^	0.64 ± 0.07 ^ab^	0.675 ± 0.06 ^b^	0.735 ± 0.05 ^ab^	0.715 ± 0.05 ^ab^	0.655 ± 0.22 ^a^
Goods coverage	99.91 ± 0.00	99.92 ± 0.00	99.91 ± 0.00	99.91 ± 0.00	99.88 ± 0.00	99.91 ± 0.00

Different superscript letters within the same row indicate significant differences among groups (*p* < 0.05).

## Data Availability

The data that support the findings of this study are available from the corresponding author upon reasonable request.
